# Can Community Health Workers in Miami Disrupt Disparities among Black People Living with HIV: A Qualitative Analysis

**DOI:** 10.2174/0118746136309444240425061403

**Published:** 2024-05-15

**Authors:** Sonjia Kenya, BreAnne Young, Lindsay Richards, Felicia Casanova, Allan Rodriguez, Jakisha Blackmon, Olveen Carrasquillo, Yue Pan, Deborah Jones-Weiss

**Affiliations:** 1 Department of Medicine, University of Miami Leonard M. Miller School of Medicine, Miami, Florida, USA; 2 Department of Public Health Sciences, University of Miami Leonard M. Miller School of Medicine, Miami, Florida, USA; 3 Department of Sociology, University of Miami Leonard M. Miller School of Medicine, Miami, Florida, USA; 4 Psychiatry & Behavioral Sciences, University of Miami Leonard M. Miller School of Medicine, Miami, Florida, USA

**Keywords:** Community health workers, HIV/AIDS, Health disparities, Black communities, PLH, Black people

## Abstract

**Aims:**

This study aims to understand how clinic-based Community Health Workers could address barriers to viral suppression and improve HIV management among Black people living with HIV.

**Background:**

South Florida is home to the greatest number of people living with HIV (PLH) in Florida, and Black communities are disproportionately impacted. Among the most promising strategies to improve HIV outcomes among Black PLH (BPLH) are Community Health Worker (CHW) interventions. Traditionally, CHWs assist PLH in non-clinical environments, and little data exists on the effects of CHW strategies in clinical settings.

**Methods:**

From March 2021 to January 2022, we administered semi-structured interviews to patients, caregivers, and clinic staff to assess their perceptions of barriers to HIV care, facilitators of HIV care, and views on CHWs using a rapid qualitative analysis framework.

**Results:**

There was significant overlap between clients and providers regarding the perceived barriers and facilitators to HIV care. Emergent themes reflected concepts surrounding HIV policy and clinic limitations, stigma across clinic- and community-based settings, and suggestions on ways CHWs can address these concerns.

**Conclusion:**

The results suggest embedding CHWs into HIV clinical teams may be an efficacious approach to address unmet social needs and overcome systemic barriers to HIV care, leading to improved care engagement and HIV outcomes among BPLH.

## 1. INTRODUCTION

Miami-Dade County is home to the highest number of people living with HIV (PLH) in Florida, and Black communities are disproportionately impacted [1, 2]. As this region experiences some of the greatest HIV disparities in the nation, it has been designated as a priority region by the U.S. Government’s strategy to End the HIV Epidemic [3].

Nearly half of Miami’s Black PLH (BPLH) are not virally suppressed, and this population represents nearly two-thirds of the County’s AIDS-related deaths [1–3]. This disparity is further pronounced from a geospatial perspective, in which clusters of local HIV infections are spatially concentrated in historically Black neighborhoods, such as Overtown (ZIP Code: 33136). In response, public health experts have called for targeted interventions to overcome care engagement and treatment adherence challenges [4].

Among the most promising strategies to improve HIV care engagement among BPLH is the Community Health Worker (CHW) model, which involves community members delivering health education and services to laypeople in vulnerable neighborhoods. Serving as the link between the community and the health system, CHWs improve rates of HIV testing and early diagnosis, help patients access care, and improve medication adherence, resulting in better health outcomes [5, 6]. CHWs also address the social determinants of HIV outcomes (housing, food security, transportation, *etc*.) that are outside a physician’s purview and traditionally work in non-clinical settings, such as patient homes or community locations (*i.e*., city parks) [7].

Research shows BPLH, even those enrolled in care, often struggle to achieve optimal outcomes [8]. Therefore, this research team has been working for the last decade to improve linkage to and retention in HIV care for BPLH through a traditional community-based CHW program. Findings from a recent initiative by the Health Resources and Services Administration HIV/AIDS Bureau, however, suggest CHWs can further address the medical and socioeconomic needs of PLH by working with physicians and other service providers as part of the clinical team [9]. While the majority of physicians support integrating CHWs into clinical care teams, there is little data on the potential of CHWs to provide effective support for HIV care in clinical environments, particularly for BPLH.

To identify gaps in care that clinic-based CHWs could potentially address for BPLH, we conducted a qualitative research study among stakeholders within our public safety-net clinic in Miami, FL. Herein, we describe the preliminary findings from this formative research and its impact on the design of our clinic-based CHW intervention.

## 2. METHODS

This research was conducted from March 2021 to January 2022 with the purpose of informing a larger future trial to assess the efficacy of clinic-based CHW intervention on HIV outcomes for BPLH. Using a rapid qualitative analysis framework, semi-structured interviews were conducted with patients, caregivers, and clinic staff to assess their perceptions of barriers and facilitators of HIV care and views on CHWs.

### 2.1. Setting & Participant Selection

All participants were recruited from the University of Miami/Jackson Health System (UM/JHS). As one of the largest academic safety-net systems in the nation, UM/JHS serves as a primary entry point for obtaining HIV care. Nearly all (95%) of Jackson’s patients identify as ethnic minorities. More than half of PLH in care at JHS are Black, and 15% are Haitian immigrants. Overall, patient satisfaction within the clinic is high, and patients with Ryan White health insurance complete an annual survey that queries satisfaction with their providers, case managers, and services offered. The most recent assessment, conducted from January to April 2021, found that over 90% were satisfied with their medical provider, with 90% stating that they were always involved in decisions about their care. Despite these high ratings, a substantial proportion of the clinic’s Black patients have not achieved optimal HIV outcomes.

To better understand the challenges to optimal HIV outcomes that CHWs may be able to address among BPLH, twenty semi-structured interviews were performed. While there are no set guidelines for estimating the sample size required for a qualitative approach, theoretical saturation, or the point at which no additional themes are generated is frequently defined in the literature by a sample size of 20 to 30 participants. As outlined by the Archives of Sexual Behavior, a sample of this size allows investigators to (1) distinguish conceptual categories of interest, (2) maximize the possibility that enough data have been collected to accurately define the relationships between themes and identify variation among participants, and (3) increases the chances that outlier cases can be explored in the data [10]. Ten patients or patient caregivers, collectively referred to as clients, were invited to participate in the interviews. Eligibility criteria for clients included self-identification as Black/African American and English-speaking, with suboptimal HIV outcomes, as indicated by an unmanaged HIV viral load (above 200 copies/mL). Potential clients were recruited from the UM/JHS Special Immunology Clinic (SIC). Prior to recruitment, the research team met with providers to select a convenience sample of clients with upcoming appointments. All eligible clients agreed to participate. Following a brief screening among clients, the clinic coordinator introduced them to a CHW, who invited the client to participate in a brief interview. Patients had no prior relationship with the CHW. To date, CHW support is not part of the standard of care, and CHW services have not been offered previously in this clinic.

The eligibility requirement for clinic staff was employment at a UM/JHS HIV clinic. The study staff initially contacted three SIC physicians and the clinic charge nurse *via* email who described the study and invited them to email the study coordinator to schedule an interview. The providers were asked at the end of the interview if they knew of any colleagues they felt would be interested in the study. The research team invited those referrals to participate *via* email. The participants were also encouraged to share the invitation email with their colleagues.

### 2.2. Measures

Conceptually guided by the Consolidated Framework for Implementation Research (CFIR), interviews assessed organizational and positional perceptions across three domains: challenges or barriers to HIV care, facilitators of HIV care, and views on CHWs and their impact as part of clinic operations [11]. Two interview guides were created: a 10-item guide for clinicians and clinic staff and a 9-item guide for patients and caregivers (Tables 1 & 2). The guides used semi-structured questions to examine barriers and facilitators to HIV care across CFIR domains, including a) intervention characteristics; b) outer setting, including the health system, HIPPAA, and regulatory issues; c) inner setting, including structural, staffing, and workload characteristics at the clinic; d) individual characteristics, including knowledge and beliefs about CHWs; and e) the implementation process, which focused on intervention engagement *via* planning and execution. Prior to finalizing the guide, all questions were reviewed and approved by the study PI, the Director of HIV clinics, the Dean of the Clinical & Translational Science Institute, and a senior faculty member who specializes in identifying barriers and facilitators to HIV care.

### 2.3. Data Collection

Qualitative interviews were conducted by research associates with advanced degrees, HIV counseling certification through the Florida Department of Health, and CHW training experience. Research associates conducted the interviews in English, and each interview lasted for an average of 45 minutes. The interviews were performed in person or *via* Zoom teleconferencing software. In-person interviews were conducted in a private SIC office, and all interviews were audio-recorded for analysis. Audio files and the interviewer’s digital notes from each session were stored on a password-protected laptop and synced to the university’s encrypted cloud-based storage system. Participants received $100 for their time *via* cash or e-transfer applications such as Venmo and CashApp.

Participants were verbally informed of the study purpose, procedures, risks, benefits, and their rights as research participants prior to the interview. Informed consent was orally obtained, and participants were provided with a physical or digital consent form upon request. Prior to the study onset, this study was approved by the University of Miami Institutional Review Board and Jackson Health Clinical Research Review Committee (IRB # 20201234).

### 2.4. Qualitative Analysis

Data were analyzed by three researchers with a range of professional qualifications, disciplinary affiliations, and training experience, including faculty and doctoral students of diverse racial and sexual minoritized identities in various academic disciplines, including nursing, psychology, and internal medicine. Two of the three analysts were not involved in the data collection process to reduce potential subjectivity in the interpretation of participant experiences. The analysts were trained in qualitative interviewing and analysis, particularly the rapid qualitative approach, by a faculty member who has trained and published on this analytic method [12].

This approach has been thoroughly described in peer-reviewed publications [13–15]. Several studies found rapid qualitative analyses to be efficient and comparable to results obtained from standard thematic analyses [12]. Aligned with this methodology, the interviews were not transcribed; rather, analysts summarized the content in one- or two pages of key points to highlight the salient information captured from each recording [13, 14]. Important quotes were the only parts of the interview transcribed verbatim. The data was divided in half between two analysts. Each analyst began with five files; once these records were summarized, an audit was performed in which each analyst reviewed the other’s work. The edits were then discussed to ensure consensus on the material.

Once the guides were finalized, the summarized data was transferred to an interview summary matrix organized by domain, including clinic- and community-based factors influencing treatment, disposition towards CHW support, and perspectives on the gaps CHWs could help address. The analysts independently identified the emerging themes before meeting to compare their findings, discuss any edits, and finalize the initial themes list. This process was repeated for the remaining records, creating an iterative development of summaries and themes.

## 3. RESULTS

Twenty interviews were conducted: ten from the client’s perspective and ten from the perspective of the clinical team. All clients identified as Black (*n*=10), with one individual identifying as Afro-Latine and one as Haitian. Nine clients were male patients living with HIV, and one was a female caregiver. The participants in the clinician interviews (*n*=10) included five medical doctors, two program managers, two case managers, and a program assistant. Eight were female, two were male, and they identified as Black (*n*=5), White (*n*=2), and Hispanic (*n*=3); three participants were Black-Caribbean immigrants (Table 1).

There was significant overlap between clients and providers regarding perceived barriers and facilitators to HIV care. The emerging themes reflected concepts surrounding HIV policy and clinic limitations, stigma across clinic- and community-based settings, and suggestions on ways CHWs can address these concerns (Table 3).

### 3.1. Facilitators of ART Adherence

#### 3.1.1. Customer Service & Compassionate Patient Care

The importance of empathy towards patients throughout the healthcare system is a common theme among clients and providers. Many clients shared positive experiences of feeling cared for by the clinic staff. They described how receiving calls from clinic staff with appointments and medication reminders facilitated their treatment. One client noted, “They keep a check on me, you know? All the time, they call me.” Several clients described their experiences with unpredictable life events – such as family loss, injuries, or sudden financial commitments – and the impact these events had on their ability to access care and remain adherent. To address these challenges, services such as housing/rent assistance, career/educational support, transportation/bus passes, childcare, and checking in with clients between visits were mentioned as ways to support clients and facilitate care.

One provider discussed the need for greater sensitivity training to create an environment with increased patience and compassion for clients. Another provider stated, “The whole clinic could be restructured to be nicer. The staffing there could be more welcoming. Communication skills in the clinics for the clinic staff could be improved.” Clients also expressed a desire to feel valued, respected, and treated as active participants in their healthcare journey by having a provider explain their rest results or asking about their day-to-day lives. One client stated, “Just be there for us. As long as [you are] there for us when we have something to say, and [you are] there to listen. You [cannot] be it all. We need somebody there by her side, not behind us, not in front of us, by our side.”

#### 3.1.2. Collaborate with Existing Structures

Clients and providers noted that existing support networks, such as patient navigators, medical assistants, and social workers are helpful in the clinic setting. For example, one clinician observed that patient navigators at the PrEP Mobile Clinic discussed care plans with clients and that project managers handled insurance and the transition between case management and the navigator team. Providers also noted that patient navigators provided moral support so clients could “find it easier to reach out if they feel like somebody else is in their corner.”

#### 3.1.3. Sources of Mental, Social, and Spiritual Support

Both clients and providers mentioned facilitating disclosure with friends/family, providing emotional support, and having access to mental health resources as facilitators of care. While clients reported few support systems that were aware of their HIV status, the majority highlighted the presence of family, friends, partners, or religious communities as positive agents in their HIV care. One client mentioned that he had been scared to tell his girlfriend about his HIV status, but the doctor helped him disclose it to her. A clinician mentioned that by helping with disclosure, the partners of patients could be linked to PrEP. Another client noted the importance of spirituality in their clinical care, stating, “[I am] bringing my spiritual problems in whatever we [are] doing right now and infuse it as one. Hopefully if [I am] talking to you, the spiritual world will hear me.” Clients also expressed the need for emotional and spiritual support to help with interpersonal issues such as grief, substance use, depression, and family trauma as challenges that can accompany an HIV diagnosis.

### 3.2. Barriers to ART Adherence

#### 3.2.1. Structural and Logistical Barriers to Accessing Clinical Care

Challenges concerning clinic location, restrictive hours of operation, and limited transportation opportunities made clinic access difficult for some clients. Many services are only offered during traditional work hours or require additional case management for approval, making it difficult for clients with full-time employment to access services. Some clients and providers highlighted long wait times and noted that clients may wait upwards of two hours before seeing the physician. Clients also expressed difficulty reaching the office by phone and difficulty remembering appointments.

Some providers felt the central location and open layout of the clinic could create a stigmatizing environment for patients, and many clients feared a confidentiality breach if observed in the clinic by a community member. As one provider explained, the perception that “people will know why [you are] there” fosters apprehension among clients and can lead to service delays andincreased risk for ART non-adherence. The physical commute to access care was also mentioned as a significant barrier. For example, certain insurance plans limit the services and pharmacies clients can utilize. As the provider explained, only one pharmacy in the county provides Ryan White recipients free medication and is about 30–45 minutes away from the clinic.

Feeling lost or overwhelmed by the healthcare system was another barrier identified by clients and providers. Clients reported difficulty following through procedures, labs, imaging, or filling prescriptions. One provider expressed concern about how many different people clients must interact within the clinic. Another provider discussed how guidelines for a federally funded program frequently changed, making it difficult to give clients accurate information on how to access their medication. Clinicians raised concerns that the plans discussed in the clinic may not be well understood by patients, and the home circumstances of patients may not be understood by providers. Clinicians were also concerned about client education regarding medication compliance or guidelines for comorbidities such as diabetes or hypertension.

#### 3.2.2. Stigmatizing Treatment & Discriminatory Experiences

Experiences surrounding staff attitudes, stigmatizing treatment from providers, and minimal trust in the healthcare system were discussed by clients and providers. One clinician believed that other providers were discriminatory towards LGBTQ clients and described witnessing another colleague publicly misgender LGBTQ clients when they came in for care. Clients reported feeling that clinical teams did not understand them and wished their providers would listen more. As one client discussed, “(the doctors) need to listen to what I got to say so they can observe me and see what the problems (I’m having) are. A lot of times, these doctors don’t listen, they only talk.” Some clients also expressed feeling their physician “talks down” or was “talking to” them as opposed to “talking with” them.

Clients also expressed that they experienced discrimination when accessing affordable care, stating that the quality of their treatment changed based on their insurance plan (private insurer *vs* Medicaid or Ryan White). One client chose to purchase another policy, despite financial concerns, to avoid prejudicial “off-remarks” from clinic staff. Another described how their provider’s behavior with patients felt akin to “treating (them) like a corpse.” Generally, many of the client interviews share theperspecti ve that the system “[does not] care about [us]”. Providers also highlighted stigma and discrimination as barriers to HIV care, although they perceived this barrier as primarily stemming from neighbors and peers in the community setting.

#### 3.2.3. Socioeconomic Barriers & Unmet Social Needs

Community barriers surrounding poverty and lack of access to social services include challenges with housing and homelessness, financial instability, child support, limited transportation, and immigration. For example, one client discussed how homelessness complicated their HIV care as they lost their medication when their bags were stolen. Providers and clients also reported difficulties obtaining or linking patients to housing programs such as Section 8. Financial obligations were noted as challenges, and one patient reported that some people sell their HIV medications to make ends meet. Lack of childcare leading to missed appointments was also expressed as a problem, especially for women.

### 3.3. Perspectives on CHW Support

All participants, including PLH, caregivers, and providers, believed CHW support in clinical and community settings could be beneficial for clients and clinic staff. As community members, CHWs often have an identity or shared experience that aligns with the communities they serve, allowing them to form a unique bond with clients as their “link to the medical establishment.” Clients and providers indicated that CHWs could serve as extensions of the healthcare system to support education, medication adherence, appointment management, and social services navigation. Other clients also noted that CHWs could improve communication during visits to better align patient and provider plans.

#### 3.3.1. CHWs can Bridge the Gaps in Access & Communication

Both clients and providers felt CHWs would be a positive addition to the clinic setting, facilitating patient care and mitigating gaps in care delivery. It was noted that during the clinic visit, CHWs could empower clients to negotiate the flow of conversation with their doctor. One client expressed interest in speaking to a CHW before and after clinic appointments to help interpret medical terminology. Providers also expressed interest in CHWs reinforcing messages discussed during the visit as well as helping clients review important information to avoid challenges within the healthcare system, such as unnecessary bills or expired referrals.

All participants noted the presence of several social barriers that directly and indirectly impeded clients’ access to care, with housing instability, job insecurity, and insurance complications reported as the most common challenges. Many participants also identified how CHWs could navigate clients through these complex processes to facilitate better access to resources. Other providers highlighted that not all patients need support from a CHW and that interventions should be targeted towards those facing challenges such as missing appointments, not taking medications, or struggling with comorbidities.

CHWs could also help bridge the gap between community and clinic visits. One provider noted, “The reality is that they [patients] do not talk much to me about what is happening in their community.” Suggestions for CHW roles included educating clients about how to do wellness checks and reaching clients through home visits where CHWs could “open the patient’s cupboards” to provide education about foods in the home. As one clinician explained, patients may be adherent to HIV medication but may need support with diet, exercise, stress reduction techniques, or other healthy habits. Providers noted that CHWs could enable clients to receive health services in non-clinical settings outside of regular working hours, such as glucose checks and blood pressure screening. Clients also expressed interest in-home visits to have someone check in with them and to improve limited healthcare access among clients with impairment or limited physical mobility.

#### 3.3.2. CHWs can be Advocates

Personal characteristics and attitudes of CHWs were also cited as traits that made them a valuable resource for clients experiencing challenging moments in their lives and healthcare situations. For instance, clients reported a belief that CHWs are good at treating patients like “regular people” and felt they were able to maintain their confidentiality. A provider also mentioned that CHWs could provide moral support for newly diagnosed PLH, highlighting the value CHWs could add to the clinical team as new patients begin their care journey.

## 4. DISCUSSION

To develop a better understanding of the clinical and community-based barriers that CHWs could address to improve HIV outcomes among BPLH, we conducted interviews with treatment nonadherent BPLH and their HIV providers at Miami’s largest public safety-net hospital. Both groups described similar factors influencing care from the clinic perspective, including concerns about structural barriers, difficulty navigating the healthcare system, stigma and discrimination, and unmet socioeconomic needs. Despite these barriers, participants noted several facilitators to improve the quality of HIV care among this population, including customer service and compassionate care delivery, collaborating with existing HIV care organizations, and sources of support for mental and emotional well-being.

The stigma associated with receiving care at HIV clinics is a well-documented barrier and was raised by most clients in the present study. Acknowledging this, many HIV providers have established mobile sites and service centers without obvious branding as an HIV treatment facility [16]. Additional strategies identified by participants include redesigning HIV clinics to create a more welcoming and destigmatizing environment for patients. Notably, patients and providers in this study highlighted the availability of CHWs as an alternative to clinic-based care. As trained HIV counselors, CHWs can facilitate clients with telehealth appointments and other forms of home-based care, providing patients the ability to access more discrete support.

HIV-related discrimination and poor treatment in healthcare settings were identified barriers, which is consistent with research showing that HIV-related discrimination is pervasive in clinical settings [17–19]. While none of the PLH included in this study indicated their discriminatory experiences prevented them from attending the clinic, it did reduce the quality and timeliness of their care and served to further stigmatize the process. Given that participants were recruited from the clinic, it is possible that others who experienced discrimination declined to return to care. As noted earlier, over 90% of clinic patients were satisfied with their care; however, this study was focused on identifying barriers to care among the minority of patients with poor HIV outcomes.

Socioeconomic challenges, such as taking off work for clinic appointments and financing transportation, were among the most prominent barriers discussed by both groups, and many providers acknowledged the potential impact CHWs could have on addressing these issues. For example, CHWs can facilitate home-based care *via* telehealth using mobile devices, eliminating the need for clients to choose between going to work or the clinic. CHWs are also skilled in facilitating the use of social services, such as accessing food stamps, housing assistance, and transportation vouchers [20–22].

As supported in other literature, this research suggests that CHWs are a powerful strategy to overcome many of the barriers that lead to suboptimal outcomes among high-risk populations living with HIV [23]. While most CHWs are community-based, a growing body of literature indicates that CHWs based in clinics can also significantly improve clinical HIV outcomes [24, 25]. As members of a patient’s care team, CHWs could bring familiarity into the clinic setting through participation in clinical team meetings, case debriefings, and patient appointments to facilitate better interaction with providers [8, 25]. This research also supports the idea that traditional community-based CHW models continue to be a valuable modality of care for addressing many of the barriers noted above.

While the authors have substantial documented experience implementing community-based CHW interventions to improve outcomes among BPLH, the main purpose of the study herein was to yield patient and provider perspectives on strategies to integrate CHWs into a clinical HIV team at South Florida’s largest safety-net hospital, in order to address the needs of patients who are enrolled in clinical care but not achieving optimal outcomes [21, 22, 26, 27]. Researchers met with clinical providers from the HIV team to share these results, including issues of stigma in care delivery and compassion. In response to participant feedback about barriers to referral care, the team worked with the clinic director on these identified barriers and explained how CHWs can help bridge the gaps.

The results from this qualitative study contribute to the growing literature on the invaluable role of CHW in the healthcare system for improving healthcare outcomes and access for disenfranchised minoritized communities [5–7, 25]. Given that this study focused specifically on BPLH in Miami, additional research is needed to determine whether similar results can be perceived in different contexts or among different populations. Potential limitations include using a convenience sample of only English-speaking participants. As a region with extensive cultural diversity and a substantial immigrant population, conducting the interviews in English alone may not have captured the full range of perspectives surrounding clinical CHWs. Another limitation includes not conducting interviews with CHWs. While CHWs were members of the research team and contributed to developing the interview guides, it would have been valuable to also address how CHWs feel about their role in this clinical setting. Additionally, no patients identifying as female were interviewed. While future studies should seek out the perspectives of Black women living with HIV, the population included in this study is consistent with HIV prevalence in Miami-Dade County, where men currently account for 76% of HIV cases in the local Black population [1, 2]. It is also possible that the participants’ responses were influenced by social desirability bias, leading them to report more favorably regarding perspectives on community health workers or the clinic experience.

## CONCLUSION

This study is one of the first to yield insights from a patient and provider perspective on the potential role of CHWs in addressing gaps in HIV care among BPLH. The findings highlight the barriers and facilitators CHWs can address to improve the Black experience in HIV care through a clinic-integrated intervention. The results of this study suggest that embedding CHW support into HIV clinical care may be a promising approach to address the identified gaps in care engagement and navigation, including unmet social needs and experiences of stigma and discrimination.

## Figures and Tables

**Table 1.** Demographics characteristics of INSTACARE qualitative interview participants from March 2021 to January 2022.

-	Clinicians	Clients
**Sex**	-	-

Men	2	9
Women	8	1

**Race/Ethnicity**	-	-

Black	5	10
− African American	2	8
− Hispanic	–	1
− Haitian/Caribbean	3	1
Non-Hispanic White	2	–
Hispanic	3	–

**Clinic Position**	-	-

Physician	5	–
Case Manager	2	–
Program Assistant	1	–
Program Manager	2	–

**Table 2.** Interview questions developed by the INSTACARE investigative team, stratified by domains from the Consolidated Framework for Implementation Research.

Domain	Client Interview Questions	Clinician Interview Questions
**Intervention**	• What are some services that you think would help make your clinic experience more beneficial? • Tell me about your experience with using a mobile phone. Do you use your phone for texting? Do you use any mobile apps?	• What are some services that you think would improve a patient’s clinic experience? • What are your thoughts on using a mobile phone app or texting service to help patients manage their care? • What do you think are other key considerations for providing HIV care and achieving viral load suppression for people in this community?
**Outer Setting**	• Thinking about your community, what are the top three challenges you face when it comes to managing your HIV?	• Thinking about your patients’ community, what are the top three barriers your patients face when it comes to managing their HIV?
**Inner Setting**	• Thinking about your time in the clinic, what are the top three challenges you face when it comes to managing your HIV? • Do you have health insurance or coverage through the Ryan White Program? If not, have you heard of this program? If so, how easy or difficult has it been to access the services offered through the program?	• Thinking about your experience with patients in the clinic, what are the top three challenges your patients face when it comes to managing their HIV?
**Individual**	• Do you have social support? For example, is there someone you can talk to, such as a friend or a family member, if you need help or support?	-
**Process**	• Would it help to have someone like a Community Health Worker at the clinic that could discuss your care plan and help you during appointments? • Would you be interested in receiving support from a CHW to help manage your HIV outside of the clinic? • What else do you think CHWs should consider providing the best support with managing HIV care and achieving viral load suppression?	• Would it help to have someone at the clinic that could further discuss care plans with the patient and help during appointments? • What are some benefits that CHWs could provide as a member of your clinical care team? • What are some services that CHWs could provide in clinic to improve care delivery? • Are there any gaps in care that CHWs can address outside the clinic? • How should we integrate CHWs into the clinical care team? What help would be most beneficial for you when treating a patient?

**Table 3.** Themes from INSTACARE interviews on clinical care for Black patients living with HIV, including example quotes from participating patients (Pt.) and providers/clinic personnel (Cl) collected from March 2021 to January 2022.

**Barriers**

“I’ve never been shown how to monitor my viral load. I don’t even know what that means. How could I know how to do that? Ain’t nobody ever told me that.” (Pt.-Interview) “Sometimes there’s not a good mechanism for the client to be notified if they’re there have no more prescriptions” – “it wasn’t processed correctly or something. And then they couldn’t get the medication. So I think systems sometimes really can be a barrier.” (Cl-Interview) “Housing is a big one. And […] if there’s a family disruption, if someone is sick or someone dies or there’s a divorce or someone gets kicked out of a house, then that can send everything into disarray.” (Cl-Interview) “The clinic and the idea of being there and being seen and people, you know, kind of knowing or the perception that people will know why you’re there” (Cl-Interview) “I had to go and purchase another insurance plan […] because with Ryan White, just nobody seems to want to give me the time of day. […] My doctor even said to me, ‘Well, we’re doing this for free’ or something like that, you know, just a little off [re]marks” (Pt.-Interview)

**Facilitators**

“So I think that part of it, it’s a community like they used to be, sometimes there’s limited resource…like for rent assistance. […] They don’t have all the funds all the time and for people that really need that, sometimes it’s not enough.” (Cl-Interview) “Even if you have the Ryan White and you go to the emergency hospital, they’re going to charge you because it’s not covered... have to pay out of your own pocket or they send you a bill.” (Cl-Interview)

**Perspectives on CHW Support**

“Just be there for us. And that makes me us feel good. Somebody care[s]. You’re not treating us like a corpse because we’re not dead.” (Pt.-Interview) “Empathy, you know, just knowing not to be so quick to judge” (Cl-Interview) “Community health workers can really help […] us recognize individual goals of care in terms of what the priorities are for those patients, because sometimes we don’t communicate that well… Our priorities for patients sometimes are not matching the patient priorities. And sometimes the patients may not feel that they can advocate for themselves.” (Cl-Interview)

## Data Availability

The data supporting the findings of the article is available in the University of Miami Scholarship@Miami Repository at library.miami.edu.

## LIST OF ABBREVIATIONS

PLH: People Living with HIV
CHW: Community Health Worker
SIC: Special Immunology Clinic
CFIR: Consolidated Framework for Implementation Research
